# Germline pathogenic variant in *PIK3CA* leading to symmetrical overgrowth with marked macrocephaly and mild global developmental delay

**DOI:** 10.1002/mgg3.845

**Published:** 2019-07-09

**Authors:** Marcella Zollino, Carlotta Ranieri, Valentina Grossi, Chiara Leoni, Serena Lattante, Daniela Mazzà, Cristiano Simone, Nicoletta Resta

**Affiliations:** ^1^ Fondazione Policlinico Universitario A. Gemelli IRCCS Unità Operativa Complessa di Genetica Medica Rome Italy; ^2^ Istituto di Medicina Genomica, Università Cattolica del Sacro Cuore Rome Italy; ^3^ Medical Genetics, Department of Biomedical Sciences and Human Oncology (DIMO), University of Bari “Aldo Moro”, Bari Italy; ^4^ Medical Genetics, National Institute of Gastroenterology ‘S. de Bellis’ Research Hospital Castellana Grotte (BA) Italy; ^5^ Department of Human and Child Health and Public Health, Center for Rare Diseases and Birth Defects Fondazione Policlinico Universitario A. Gemelli IRCCS Rome Italy; ^6^ Istituto di Pediatria, Università Cattolica del Sacro Cuore Roma Italia

**Keywords:** germline variant, macrocephaly, overgrowth, PI3K/AKT/mTOR pathway, *PIK3CA*

## Abstract

**Background:**

Activating pathogenic variants in *PIK3CA* gene usually occur at a mosaic status and underlie a variety of segmental overgrowth phenotypes. Germline variants in *PIK3CA* have been rarely reported, described in a total of 12 patients with macrocephaly to date. Clinical and prognostic features of these germline variants have not been described in detail yet.

**Methods:**

Targeted deep sequencing by custom panel of the 21 genes involved in the PI3K/AKT/mTOR pathway was performed in a 13‐year‐old boy with macrocephaly and physical overgrowth. PI3K/AKT/mTOR pathway analysis was performed in fibroblasts by Western blot. The effects of miransertib (AKT inhibitor) and rapamycin (mTOR inhibitor) were assessed.

**Results:**

A de novo pathogenic variant (c.1090G>C; p.Gly364Arg) in *PIK3CA* gene was detected in a non‐mosaic status in peripheral blood cells, buccal smears, and skin fibroblasts. Increased levels of phosphorylated AKT residues were observed in fibroblasts, rescued by miransertib.

**Conclusion:**

Germline variants in *PIK3CA* are associated to a mild phenotype characterized by overgrowth, severe macrocephaly, mild intellectual disability, and few dysmorphic features. Investigations of PI3K/AKT/mTOR pathway should be performed in patients with severe macrocephaly and unspecific physical overgrowth. Longitudinal studies to assess prognosis and cancer predisposition are recommended.

## 1 INTRODUCTION

Somatic variants of *PIK3CA* (OMIM: 171834) are associated with segmental overgrowth disorders, which are now called *PIK3CA‐related overgrowth spectrum* (PROS) including Fibroadipose (and bone) hyperplasia or overgrowth (FAO), Hemihyperplasia Multiple Lipomatosis (HHML), Congenital Lipomatous Overgrowth, Vascular Malformations, Epidermal Nevi, Scoliosis/Skeletal and Spinal (CLOVES) syndrome; Klippel‐Trenaunay syndrome (KTS) and Megalencephaly‐Capillary Malformation Polymicrogyria (MCAP) syndrome (Keppler‐Noreuil et al., 2015). Germline variants in *PIK3CA* are reported only in 12 individuals so far (Orloff et al., 2013; Rivière et al., 2012; Yeung et al., 2017). Of importance for surveillance, the true relevance of *PIK3CA* germline variants, compared to somatic variants, in term of cancer predisposition, is currently unknown. Herein, we describe a patient with macrocephaly and mild developmental delay in whom the c.1090G>C (p.Gly364Arg) pathogenic variant in *PIK3CA* was detected in different tissues in a non‐mosaic status.

## 2 MATERIALS AND METHODS

### 2.1 Editorial policies and ethical considerations

This study was approved by the local Ethics Committee and written informed consent was obtained from the mother of the patient.

### 2.2 Patient

It is a 13‐year‐old boy born as only child from healthy non‐consanguineous parents. He was born at 38 weeks of gestational age. Maternal preeclampsia was detected in the last month. Growth parameters at birth were: weight 3,880 g (+2.5 Standard deviation (*SD*)), length 54 cm (+3 *SD*), and Occipital frontal circumference 38.5 cm (+4.5 *SD*). No facial dysmorphisms were evident at birth. He showed mild respiratory distress, treated with oxygen support in incubator for few hours, transient hypoglycemia, requiring early oral feeding, and jaundice, treated with phototherapy.

Normal acquisition of milestones was reported with exception of mild delay in independent walking. The patient hold up the head at 4 months, he sat unsupported at 7 months, and began walking independently at age 18 months. He started speech therapy at the age 3 years due to language delay. The first formal neuropsychological test was assessed at 11 years (WISC IV), showing a total IQ of 55, with language subitem being the worst skill. He attended the secondary school with support. He has fluent language, can read and write well. He is quiet and sociable, no behavioral problems and Autism spectrum disorder were reported. He never had seizures. As a child, at age 2 years, he suffered from viral infection with myocarditis and transient hepatosplenomegaly that spontaneously resolved after 6 months. Periodic ultrasound examination of heart, kidneys, and thyroid gave normal results. Endocrine evaluations, including Follicle‐stimulating hormone, Luteinizing hormone, Thyroid‐stimulating hormone, FT4‐FT3, testosterone, and IGF1, gave normal results. Kidney and liver function were normal. Growth profile in term of weight and height was always harmonic and above normal ranges for Centers for Disease Control and Prevention's growth charts (respectively, weight at +3.5 *SD* and height at 4 *SD*), whereas Occipital frontal circumference measure progressively increased till +8 *SD* at the age of 10 years. A bone age study was performed at 7 years of age, showing mild advanced bone age (8 years). At age 12 years, weight was 80 kg (+3.6 *SD*), height 180 cm (+4 *SD*), Occipital frontal circumference 66.5 cm (+8 *SD*).

**Figure 1:**
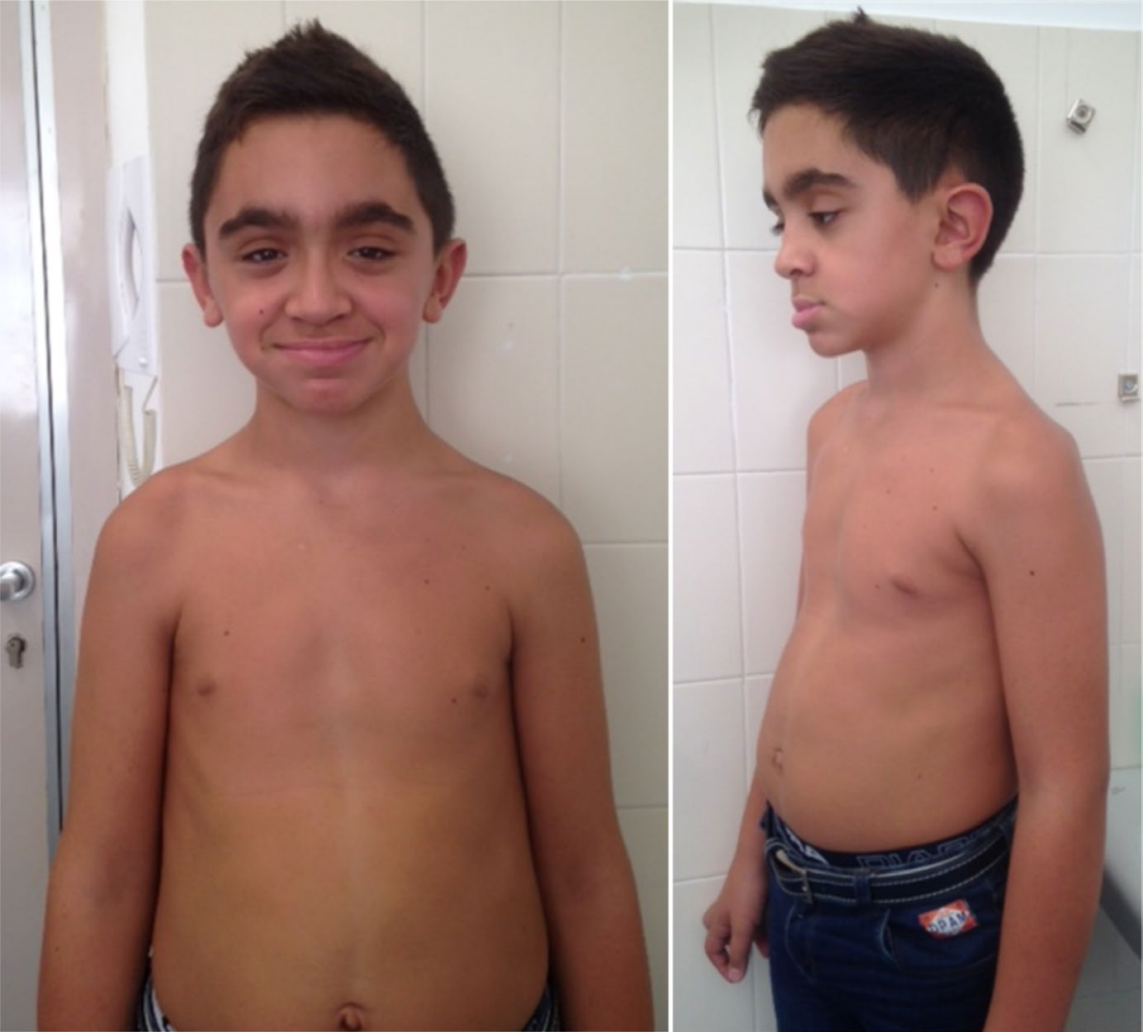
Frontal and lateral view of the patient at age 11 years. Please note arched and bushy eyebrows, palpebral ptosis, thin and elongated nasal bridge, morsus inversus with prominent lower lip, large and anteverted ears

He was presented with trunk hypotonia and minor facial anomalies (Figure 1). Pectus excavatum, mild joint hyperlaxity (Beighton score 3/9), and moderate scoliosis treated with corset, were also present. He underwent dermatological examination and dermoscopy because of multiple small acquired nevi. The only atypical lesion was surgically excised, and diagnosed as dysplastic Clark nevus. A small hyperkeratotic lesion on the foreskin of about 3 mm in diameter was present from birth. Brain CT scan performed at age 2 years, due to macrocephaly, showed hypodensity of the bilateral posterior periventricular white matter, and mild asymmetric lateral ventricle enlargement. Brain MRI at 12 years showed a Chiari malformation type I with right cerebellar tonsillar ectopia 12 mm below mcRae's line and mild lateral ventricular asymmetry without hydrocephalus. Symmetric megalencephaly without polymicrogyria was diagnosed.

### 2.3 Methods

The patient underwent array‐CGH (Agilent Technologies) and *PTEN* analysis (sequencing and MLPA), with normal results. Targeted deep sequencing by custom panel of the 21 genes involved in the PI3K/AKT/mTOR pathway was performed according to our previous report (Loconte et al., 2015; Ranieri et al., 2018). Results were confirmed by Sanger sequencing. Patients‐derived primary fibroblasts were obtained from a normal skin area and grown according to our previous report (Loconte et al., 2015; Ranieri et al., 2018). PI3K/AKT/mTOR pathway analysis was performed by Western blot as described (Ranieri et al., 2018). The effects of PI3K/AKT/mTOR pathway inhibitors, miransertib (AKT inhibitor) and rapamycin (mTOR inhibitor), were assessed.

## 3 RESULTS AND DISCUSSION

**Figure 2:**
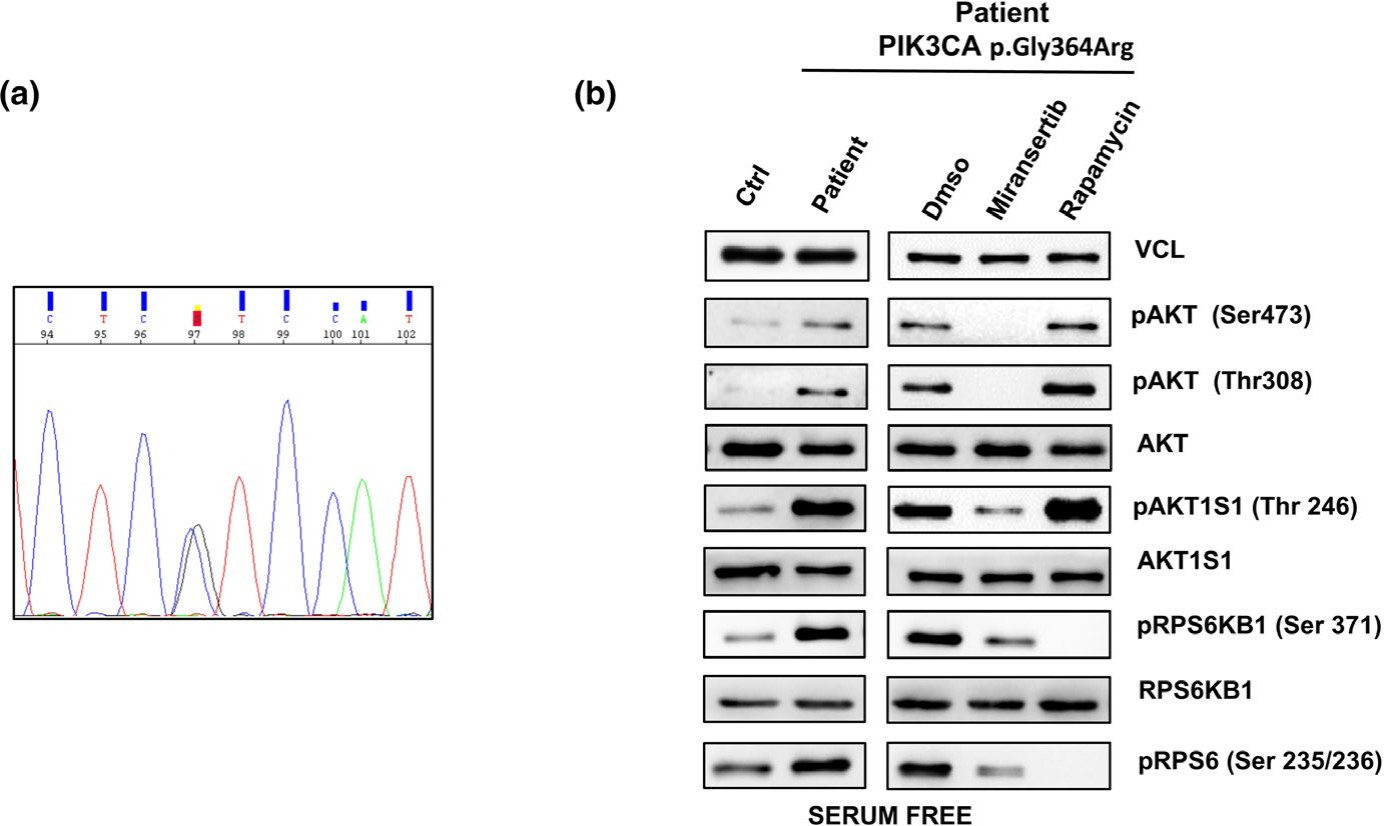
AKT activation in patient's primary fibroblasts. (a) Sanger sequencing validation of the c.1090G>C (p.Gly364Arg) variant in the *PIK3CA* gene detected by targeted deep sequencing. (b) Primary fibroblasts (from biopsies of a healthy control and the patient) were cultured in the absence of serum. Immunoblot analysis was performed to evaluate PI3K/AKT/mTOR pathway. Miransertib counteracts overactivation of PI3K signal pathway in mutant cells and the effect of Miransertib is different from rapamycin in primary fibroblasts. Primary fibroblasts from biopsies of a patient were treated with Miransertib (1 μM) in the absence of serum and immunoblot analysis was performed to evaluate pAKT (Ser473), pAKT (Thr308), total AKT, pAKT1S1 (Thr246), total AKT1S1, pRPS6KB1 (Ser371), total RPS6KB1, and pRPS6 (Ser 235/236). Vinculin was used as total lysate controls. The presented results are representative of at least three independent experiments

We report on a new patient with a germline‐activating variant in *PIK3CA* who presented with great macrocephaly and overgrowth and with mild intellectual disability, but with very few additional morphogenic anomalies. The pathogenic variant c.1090G>C; p.Gly364Arg in *PIK3CA* (NM_006218.4) was detected in a non‐mosaic status in peripheral blood cells first, and then on buccal smears and cultured skin fibroblasts, with a frequency of about 50%–50.4% in all tissues analyzed (Figure 2a). This variant was not detected in peripheral blood lymphocytes of either parent. We evaluated the phosphorylation status of AKT (Ser473 and Thr308) and its downstream target, pAKT1S1 (Thr246), pRPS6 (Ser235/236), pRPS6Kβ1 (Ser371), in the patient's primary fibroblasts cultured in serum‐free medium primary fibroblasts. Patient's fibroblasts showed increased levels of phosphorylated AKT residue (Serine 473), which is targeted by the TORC2 complex (mTOR/Rictor) in a PI3K‐dependent manner, and of another AKT residue (Threonine 308), which is targeted by PI3K in a direct or indirect (through PDK1) manner. Moreover, mutant fibroblasts had increased phosphorylation of AKT and its downstream targets. To ascertain whether inhibition of AKT can counteract the overactivation of the PI3K/AKT/mTOR signaling, we treated patient's fibroblasts with the AKT inhibitor, miransertib. Phosphorylation of AKT and its downstream targets was significantly reduced in mutant cells (Figure 2b). The mTORC1 inhibitor, rapamycin, was observed to inhibit phosphorylation of RPS6KB1 and RPS6 but not AKT. Furthermore, rapamycin led to suppression of mTOR downstream targets greater than miransertib. Inhibition of mTORC1 signal by Rapalogs (e.g., sirolimus) was reported to activate AKT signal through a feedback mechanism (Ranieri et al., 2018).

We made this assessment in order to plan therapeutic interventions if suggested by a changing phenotype. Miransertib is an experimental, orally bioavailable, and highly selective AKT inhibitor currently tested in phase I–II trials for cancer and PS (NCT02594215, NCT03317366, NCT03317366, NCT02476955, NCT01473095).

Activating variants in *PIK3CA* usually occur at a mosaic status and underlie a variety of segmental overgrowth phenotypes. Germline variants in *PIK3CA* have been reported in a total of 12 individuals with macrocephaly (Orloff et al., 2013; Rivière et al., 2012; Yeung et al., 2017). They were detected in association with Cowden or Cowden‐like syndrome in eight patients, aged from 27 to 72 years, who also presented with true malignancies or benign tumors affecting breast, thyroid, endometrium, and kidney, and with trichilemmoma, skin or oral papilloma, lipoma, and cutaneous hemangiomas (Orloff et al., 2013). However, complete phenotypic details are not provided for these patients.

Other three patients, aged 6, 16, and 12 months, respectively, were described in the first report by Rivière et al., (2012) They were presented with great macrocephaly (3/3), overgrowth with or without asymmetry (2/3), vascular malformations (3/3), syndactyly (2/3), polydactyly (1/3), connective tissue dysplasia (2/3), hydrocephalus/ventriculomegaly (2/3), cerebellar tonsillar ectopia (2/3), and polymicrogyria (3/3).

The last patient is a 1.5‐year‐old male presented with a moderate global developmental delay, mild 2/3 syndactyly, and with brain abnormalities including megalencephaly, polymicrogyria, ventriculomegaly, and periventricular white matter signal differences (Yeung et al., 2017). Available data of all patients, including the present one, are summarized in Table 1.

**Table 1:** Clinical presentations of all reported patients harboring *PIK3CA* germline variants

	Rivière (2012) (pat LR06‐220)	Rivière, 2012 (pat LR11‐153)	Rivière, 2012 (pat LR11‐230)	Yeung et al. (2017) (pat 2)	Orloff et al. (2013) (tot 8 patients)	Total	Present case
*PIK3CA* variant	p.Arg88Gln	p.Glu453del	p.Gly1049Ser	p.Val344Met	p.Gly118Asp p.Glu135Lys p.Glu218Lys p.Val356Ile p.Arg382Lys p.Glu545Ala+ p.Ser553Thrfs∗7 p.Leu632		p.Gly364Arg
Macrocephaly	+	+	+	+	7/8	11/12	+
Polymicrogyria	+	+	+	+	n.a.	4/4	−
Cerebellar tonsillar ectopia	−	+	+	n.a.	n.a.	2/3	+
Overgrowth	−	+	+	n.a.	n.a.	2/3	+
Vascular anomalies, skin hemangiomas included	+	+	+	n.a.	2/8	5/11	−
Syndactyly/polydactyly	−	+	+	+	n.a.	3/4	−
Connective tissue dysplasia	−	+	+	n.a.	n.a.	2/3	−
Skin anomalies^a^	n.a.	n.a.	n.a.	n.a.	4/8	4/8	−
General developmental delay and language delay	n.a.	n.a.	n.a.	+	n.a.	1/1	+

Abbreviation: n.a. not available.

a Trichilemmoma, papilloma, lipoma, oral fibroma.

The most prevalent clinical manifestations in our patient were marked macrocephaly, tall stature, and mild intellectual disability. Additional features were limited to slight brain ventricle asymmetry and Chiari malformation type I defined by cerebellar tonsillar ectopia.

Study of publications suggests that only part of the features in the spectrum of the clinical phenotype described so far in association with germline variants in *PIK3CA*, especially concerning tumors, can be reasonably considered age‐related.

At present, no epidemiological data are available to offer reliable cancer risk evaluation and prognosis. Suggested by the evidence that somatic gene variants in this pathway are highly involved in cancer, we started in our patient yearly anticancer surveillance by ultrasound investigations of thyroid, kidney, and liver, and by dermatological examinations. Colonoscopy was planned from 35 years, in the absence of symptomatic features.

In an attempt to search for specificity of germline *PIK3CA* variants versus somatic variants, we reviewed the currently available population data in either fields, and we found that 41% (5/12) of the germline variants affected the C2 protein domain. On the contrary, only 6% (852/13969) of somatic variants were in the same C2 domain, as reported in the Catalogue of Somatic Mutations in Cancer (Tate et al., 2019). The variant found in our patient is here reported for the first time to be germinal in origin. In considering the somatic counterpart in tumors, COSMIC reports a few cancer cases with the C.1090G>A at the same position.

In the present report, we comparatively evaluated the clinical phenotypes associated with *PIK3CA* germline variants reported so far. Larger cohorts of patients and longitudinal studies are needed to assess nosology of these conditions, and, importantly, to define cancer risk and appropriate anticancer surveillance.

## CONFLICT OF INTEREST

None declared.
